# Impact of COVID-19 Pandemic on the Implantation of Intra-Cardiac Devices in Diabetic and Non-Diabetic Patients in the Western of Romania

**DOI:** 10.3390/medicina57050441

**Published:** 2021-05-03

**Authors:** Silvius Alexandru Pescariu, Cristina Tudoran, Gheorghe Nicusor Pop, Sorin Pescariu, Romulus Zorin Timar, Mariana Tudoran

**Affiliations:** 1 Department VI, Cardiology, University of Medicine and Pharmacy “Victor Babes” Timisoara, E. Murgu Square, Nr. 2, 300041 Timisoara, Romania; alex.pescariu@yahoo.com (S.A.P.); pop.nicusor@umft.ro (G.N.P.); pescariu.sorin@umft.ro (S.P.); 2 Department VII, Internal Medicine II, Discipline of Cardiology, University of Medicine and Pharmacy “Victor Babes” Timisoara, E. Murgu Square, Nr. 2, 300041 Timisoara, Romania; tudoran.mariana@umft.ro; 3 County Emergency Hospital, 300041 Timisoara, Romania; 4 Center of Molecular Research in Nephrology and Vascular Disease, Faculty of Medicine, University of Medicine and Pharmacy “Victor Babes” Timisoara, E. Murgu Square, Nr. 2, 300041 Timisoara, Romania; timar.romulus@umft.ro; 5 County Emergency Hospital “Pius Brinzeu”, L. Rebreanu Str., Nr. 156, 300041 Timisoara, Romania; 6 Department VII, Internal Medicine II, Division of Diabetes and Matabolic Diseases, University of Medicine and Pharmacy “Victor Babes” Timisoara, E. Murgu Square, Nr. 2, 300041 Timisoara, Romania

**Keywords:** COVID-19 pandemic, pacemaker implantation, resynchronization therapy, atrioventricular block, sick sinus syndrome, type 2 diabetes mellitus

## Abstract

*Background and Objectives*: COVID-19 pandemic severely impacted public health services worldwide, determining a significant decrease of elective cardiovascular (CV) procedures, especially in patients with associated chronic diseases such as diabetes mellitus (DM). *Materials and Methods*: This study was first started in 2019 in the western of Romania, to analyze the differences regarding the implantations of intra-cardiac devices such as permanent pacemakers (PPM), cardiac resynchronization therapy (CRT), or implantable cardioverter-defibrillators (ICD) in 351 patients with and without DM and the situation was reanalyzed at the end of 2020. *Results*: of the first 351 patients with and without DM. 28.20% of these patients had type 2 DM (*p* = 0.022), exceeding more than twice the prevalence of DM in the general population (11%). Patients with DM were younger (*p* = 0.022) and required twice as often CRT (*p* = 0.002) as non-diabetic patients. The state of these procedures was reanalyzed at the end of 2020, a dramatic decrease of all new device implantations being observed, both in non-diabetic and in patients with type 2 DM (79.37%, respectively 81.82%). *Conclusions*: COVID-19 pandemic determined a drastic decrease, with around 75% reduction of all procedures of new intra-cardiac devices implantation, both in non-diabetics, this activity being reserved mostly for emergencies.

## 1. Introduction

As the infection with SARS-CoV-2 spread worldwide, giving rise to the largest pandemic of the last centuries–COVID-19, health systems were globally overwhelmed [1,2,3]. Priority was granted to hospitals for infectious diseases, emergency services, intensive, or intermediate care units that carry the greatest burden, but several hospital wards have been completely converted to COVID units, to deal with the increasing number of patients [4,5]. The majority of the remaining services, including cardiology, have reorganized their spaces and medical teams to treat, but also to isolate, potentially SARS-CoV-2 infected patients. Since economic resources were allocated especially for specific therapies, necessary for patients with COVID-19 and personal protective equipment, and trained personal was redirected, elective cardiac assessments and therapies were severely impacted. Several scientific papers debate over the drastic reduction of procedures in catheterization laboratories [1,6,7]. According to the European Society of Cardiology suggestions, in its guide referring to the diagnosis and management of cardiovascular (CV) disease during the COVID-19 pandemic, cardiac device implantation procedures should be postponed, but several situations are not covered by these recommendations [3,8]. Another important factor is the reduced addressability of patients due to restrictive measures, lack of information, and especially due to fear of contamination with COVID-19 [9,10].

In this study, we analyzed the situation of intra-cardiac devices implantation one year after the outbreak of COVID-19 pandemic in the electrophysiology laboratory of the Institute for CV Diseases of Timisoara, responsible for all cardioverter-defibrillators (ICD) or resynchronization therapy (CRT) and for the majority of pacemaker (PM) implantations in the western of Romania. On 28 February 2020, when the COVID-19 pandemic started in our town, a study on the particularities of intra-cardiac device implants in diabetic and nondiabetic patients, that was underway in this unit, was abruptly interrupted. Because CV disease and type 2 diabetes mellitus (DM) represent two increasingly frequent and related diseases associated with high morbidity and mortality, the study was resumed after a year of interruption, the actual situation of implants being re-analyzed, in comparison to the state of the previous two years, 2018 and 2019.

While the susceptibility of diabetic patients to develop supraventricular arrhythmias, but also ventricular ones is a well-known fact in the medical literature, the prevalence of conduction disorders in this population was less analyzed [11]. Several studies indicated that patients with DM are more likely to develop cardiac conduction disturbances, such as high-degree atrio-ventricular block (AVB), intraventricular blocks, especially RBB, or sick sinus syndrome (SSS) being more likely to require PM [11]. On the other hand, among patients with cardiac pacemakers, there is a statistically significant predominance of individuals with type 2 DM, suggesting diabetes-induced impairment of the endogenous conduction system of the heart [12]. Another important aspect is the increased prevalence of dilated cardiomyopathy (DCM) associated with important systolic dysfunction and a high risk of ventricular arrhythmias resulting in sudden cardiac death (SCD) [13,14]. In order to save their lives and/or to improve their quality of life, these patients often require the implantation of ICDs or CRTs [15,16,17].

This study aims to highlight the impact of COVID-19 pandemic on the procedures of intra-cardiac-devices implantation in patients with and without type 2 DM, in a cardiovascular tertiary center during 2020 in comparison with the activity of the same unit from 01 January 2018 to 31 December 2019. Another aim was to evidence the prevalence of type 2 DM among all 386 patients admitted during 2018–2020, for the implantation of new intra-cardiac devices or carriers of devices supposed to have dysfunctions.

## 2. Materials and Methods

### 2.1. Study Group

Of all patients admitted in a cardiovascular tertiary center, by using the International classification and statistic of diseases (ICD-10-AM)–code Z95.0, we selected from the hospital’s database 351 patients suffering from various CV diseases and requiring either the implantation of a new intra-cardiac device for rhythm control or resynchronization therapy or carriers of a dysfunctional device who required hospitalization, admitted between 1 January 2018–31 December 2019 in contrast with only 35 identified to have been admitted for the same pathology during 2020. All patients’ personal data were anonymized and the Ethics Committee of our hospital approved this study, Nr. 4052/19.06.2020.

### 2.2. Methods

Each hospitalization was registered depending on the admission and discharge date and contained information on the primary diagnosis and, if applicable, one or more secondary diagnoses defined by the International Classification of Diseases–the 10th revision (ICD-10), on the medical procedures performed during the hospitalization–also coded–and on the pharmacotherapy. Patients were identified according to their unique registration number and their medical data were searched in the hospitalization records and also in the national diabetes registry for the presence or absence of DM. Additional information regarding the staging, complications, and therapy of DM were obtained from the regional diabetes registry.

### 2.3. Data Analysis

It was performed using SPSS v.25.0 (Statistical Package for the Social Sciences, Chicago, IL, USA) for Linux Mint 19. Continuous variables were presented as a mean and standard deviation (SD) or median and associated quartiles (Q1-25 percentage quartile, Q3-75 percentage quartile), and categorical data were presented as counts (percentages). The bias-corrected and accelerated (BCa) bootstrap interval (1000 bootstrap samples) was used to calculate the 95% confidence interval. We performed descriptive and inferential statistical analysis to summarize the characteristics of the study population. The results of the Shapiro-Wilk normality test showed a non-Gaussian distribution, which is why we continued to use nonparametric tests. To evaluate the prevalence of DM, bradyarrhythmias, pacemaker implantation, resynchronization therapy, and implantable cardioverter in groups, we applied the chi-squared test (χ^2^) and Fisher exact test (Freeman-Halton extension), and to compare differences in new PM implants we employed a z-test. A *p*-value of less than 0.05 was considered to indicate a statistical significance.

## 3. Results

Taking into account that our study was realized in two stages, we analyzed initially, a group of 351 patients, 221 men, and 130 women, mean age 68.91 ± 11.65, hospitalized in a tertiary cardiovascular center during 2018–2019 for intra-cardiac device-related pathology.

Subsequently, in 2020, after a year since the outbreak of COVID-19 pandemic, we identified 35 other patients hospitalized for the same pathology, 24 men and 11 women, mean age 66.37 ± 13.5 years.

**Figure 1:**
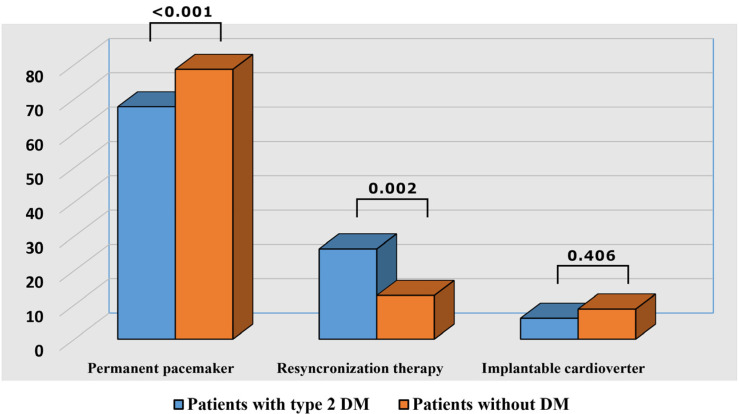
Distribution of intra-cardiac devices in patients with and without diabetes mellitus in 2018 and 2019. Legend: diabetes mellitus—DM.

Of all the initial 351 patients, 265 had pathology related to PM: 109 suffered the implantation of a new PM, 83 had dysfunctions of the existing one–mostly depleted battery needing replacement–and 73 were PM carriers hospitalized for other complications. The prevailing indication for PM implantation was AVB, followed by SSS and slow atrial fibrillation (AF) and only 7 with carotid sinus hypersensitivity (CSHS). Other 58 patients required CRT for dilated cardiomyopathies of various etiology and chronic heart failure with reduced ejection fraction: 21 had new implantations and 37 were carriers, 12 of them with a depleted battery and 25 with other cardiovascular problems–mostly decompensated heart failure (HF). The remaining 28 patients had implantable ICDs for ventricular arrhythmias or were survivors of SCD: 7 new cases and 21 carriers see Table 1, Figure 1.

By contrast to this situation, during 2020, only 35 patients were admitted for intra-cardiac devices related pathology. Of all these 35 patients, 9 (25.71%) had type 2 DM, male gender prevailed and they were younger than those without diabetes, characteristics similar to our findings before the pandemic. The majority of cases 26 (74.28%), were admitted for PM related pathology, most of them 19 patients, needing the implantation of a new device, other 6 which had depleted batteries, requiring replacement, and 1 with decubitus.

Of all 351 patients admitted during 2018–2019, with intra-cardiac devices, 99 (28.20%) were diagnosed with type 2 DM. This prevalence is considerably higher than the prevalence of type 2 DM reported in our country (11% according to Predatorr study). Male gender prevailed (63.63%) and they were younger (mean age 66.65 ± 9.76) than those without DM (*p* = 0.022), see Table 1, Figure 1. Of these patients, 67 (67.67%) were hospitalized due to PM pathology: 26 (38.8%) requiring the implantation of a new device for various bradyarrhythmias–the most frequent was AVB, followed by SSS. Although subjects without DM significantly outnumbered those with diabetes (*p* < 0.001), the latter had a significantly higher indication for dual-chamber PM. According to our results, there were no statistically significant differences neither concerning the main indication for permanent PM (PPM) implantation nor the number of new implants between patients with and without type2 DM, Table 1, Figure 1. The remaining patients had either dysfunctions of the existing PM (in the majority of cases battery replacement) or other CVD complications.

Concerning the indication for CRT in our study group, it was twice more frequent in diabetic patients (26.26% vs. 12.69%, *p* = 0.002), an aspect that was expectable taking into account the increased prevalence of DCM in this category of population, see Table 1 and Figure 1.

The third type of intra-cardiac device analyzed in our study were ICDs. The number of patients with life-threatening ventricular arrhythmias or survivors of SCD receiving this therapy was less numerous and there were no significant differences between diabetic and non-diabetic patients (*p* = 0.406), possibly due to the increased number of CRTs implanted in the first group.

**Figure 2:**
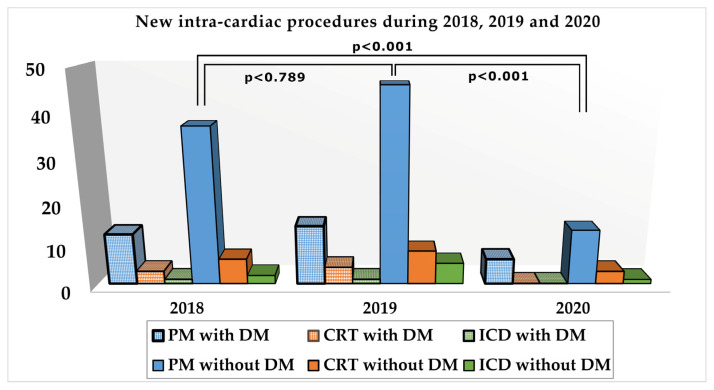
New intra-cardiac devices implantations in patients with and without diabetes mellitus. Legend: diabetes mellitus—DM; pacemaker—PM; resynchronization therapy—CRT; implantable cardioverter—ICD.

All patients were admitted as emergencies, 12 with third-degree AVB, 11 with severe bradycardia due to SSS, 2 with slow atrial fibrillation, and one with repeated syncope due to CSHS. It was a dramatic decrease, with 74.46% in non-diabetics and with 76.2% in patients with DM, of this type of procedure compared to the mean of the preceding two years. It was the principal intervention performed in diabetic patients (88.88%). Referring to resynchronization therapy, only 3 new procedures were performed in non-diabetic patients with dilated cardiomyopathy and reduced ejection fraction, representing a reduction of 57.15% in subjects without DM and with 100% in those with type 2 DM in comparison with the mean of previous years. About implantable cardioverter, 5 patients were admitted for events related to ICDs and only one, without DM, for new implantation indicating a drastic reduction of this procedure, Table 1, Figure 2).

## 4. Discussion

During the outbreak of COVID-19 pandemic, a massive reduction of emergency presentations and non-COVID pathologies were reported worldwide. As this trend continued further, in periods with a lower number of COVID-19 patients and we cannot assume that such a decrease of cases with myocardial infarction, acute coronary syndrome, stroke, and other non-COVID pathology is real, there are alternative explanations for these aspects. Apart from health politics, re-location of economic resources and shortage of personal, a reduced addressability of patients has been observed. This phenomenon is explained partially, by the fear of contamination with the SARS-CoV-2 virus, but there are other contributing factors: difficulties in scheduling medical consultations or getting to health care providers, associated with other psychosocial causes. The most affected were elderly people, with concomitant chronic disease, especially if they were requiring elective CV procedures as the implantation of an intra-cardiac device. Although there are guidelines for the electrophysiology procedures [8], some studies debate over difficulties and severe reduction of the intra-cardiac device implantations [18,19]. In our study, that was started initially, as a five years analysis of differences regarding the implantation of intra-cardiac devices in subjects with and without type 2 DM, when we wanted to resume the study, we noticed important differences in the categories of patients in which intra-cardiac devices were implanted during 2020, so we continued to research the impact of the COVID-19 pandemic on these procedures in both categories of patients. Preliminary results for 2018 and 2019 had indicated a double prevalence of intracardiac device implants in diabetic patients compared to non-diabetics, who were older than the first. Furthermore, in patients with diabetes the number of CRT implants was double compared to those without diabetes. Type 2 DM is also an important factor implicated in the physiopathology of coronary artery disease (CAD), myocardial infarction (MI), and congestive heart failure (CHF). The increased prevalence of arrhythmias and conduction disturbances in diabetic patients is a well-known entity debated in many studies [12,15,16,17]. According to some statistics, the most frequent arrhythmia in the diabetic population is sinus tachycardia, detected in 32% of patients, followed by AVB (in 20%), sinus bradycardia and atrial fibrillation (each in 15%), premature ventricular contractions (10%) and paroxysmal supraventricular tachycardia and ventricular tachycardia (1%) responsible for sudden cardiac death [13,14,17]. There is some evidence that conduction disturbances like third-degree atrioventricular block (AVB) and right bundle branch block (RBB), generally attributed to idiopathic fibrosis of the conduction system, are more common than expected in patients with DM [12,20]. Other responsible pathophysiological factors are CAD, diabetic microangiopathy or increased cholinergic sensitivity [13,15,16,21,22]. Dalgaard et al. analyzed in their study, several Danish medical databases for patients with PPM implantation during 2001 and 2012 and evidenced an increased prevalence of DM among them (16.8%–*p* < 0.001) [14].

In our comparative study regarding the reduction of intra-cardiac device implantation during the COVID-19 pandemic, we highlighted a significant decrease of all types of elective procedures in both categories of patients. But, while the decrease of new pacemaker implantations was around 75% in both, non-diabetic and diabetic patients, thus higher (74.4% versus 76.2%) in the last category, there was a tremendous difference regarding the reduction of new CRTs and ICDs implantations in diabetics, which was of 100%. That was a very surprising finding because, before the COVID-19 pandemic, the number of CRT procedures in patients with type 2 DM was double than in non-diabetics, an aspect explained by the high prevalence of dilated cardiomyopathy (DCM) in the first group. Many studies highlighted the association between DM and DCM, as well as pathophysiological mechanisms responsible for its occurrence. DCM and vascular dysfunction are results of myocardial fibrosis and inflammation, which are, therefore, key determinants of cardiac dysfunction [21,22]. Furthermore, CAD and systolic dysfunction are considered to be produced by alterations in oxidative stress and hyperglycemia [21,22,23]. Often patients suffering from CHF with impaired systolic function and severe hemodynamic and electrical disturbances were likely to receive CRT therapy or to require the implantation of ICD to prevent SCD in those with ventricular arrhythmias.

It is known that advanced age, diabetes, obesity, and CV diseases were the worst prognostic factors for fatal outcomes in COVID-19. We assumed that the considerable reduction of the number of intra-cardiac devices procedures, both, in patients with and without DM, during the COVID-19 pandemic could be explained by multiple factors, as difficulties to arrive at the hospital, to schedule their medical controls and, especially, the fear of being infected with the SARS-CoV-2 virus in the hospital, as suggested also by other authors [6,9,24,25]. Another hypothesis, that could justify, at least partially, the dramatic decrease of elective procedures in diabetic patients is that considering themselves more vulnerable to COVID-19, they have underestimated or ignored their symptoms and delayed the presentation to the hospital. All these hypotheses explain the deleterious impact of COVID-19 pandemic on population health, being responsible for a supplementary increase in morbidity and mortality.

## 5. Conclusions

COVID-19 pandemic determined a dramatic decrease of intra-cardiac devices related procedures, which were mostly limited to emergency pacemaker implantations. Diabetic patients which are predisposed to develop dilated cardiomyopathy and/or cardiac arrhythmias, requiring more frequently and at a younger age therapy based on intra-cardiac devices, were particularly affected by the reduction of elective cardio-vascular procedures during the COVID-19 pandemic, and that, because of the fear of infection with SARS-CoV-2 virus, they avoided medical services and/or ignored their symptoms.

## Figures and Tables

**Table 1:** Characteristics of patients with intra-cardiac devices in 2018, 2019 and 2020.

Characteristics	Patients with DM			Patients without DM			*p*
	2018–44P	2019–55P	2020–9P	2018–110P	2019–142P	2020–26P	
Male gender	29–65.9%	34–61.81%	5–55.55%	69–62.72%	89–62.69%	19–73.07%	NS
Mean age (years)	67.27 ± 10.3	66.65 ± 9.76	64 ± 6.22	69.35 ± 13	69.8 ± 12.22	67.53 ± 12	0.022
Pacemakers:	32–72.72%	35–63.63%	8–88.88%	88–80%	110–77.46%	18–69.23%	<0.001
single-chamber	15–46.87%	16–45.71%	4–50%	55–62.5%	54–%	10–55.55%	NS
dual-chamber	17–53.12%	19–54.28%	4–50%	33–37.5%	56–%	8–44.44%	NS
New implants	12–37.5%	14–40%	6–75%	37–42.04%	46–%	13–72.22%	NS
Main indication for pacemaker:							
AVB	16–50%	20–57.14%	3–37.5%	39–44.31%	65–52.52%	9–50%	NS
SSS	8–25%	11–31.42%	3–37.5%	24–27.27%	28–26.26%	8–44.44%	NS
CSHS	1–3.12%	-	-	3–3.4%	3–3.03%	1–5.55%	NS
Slow AF	7–21.87%	4–11.42%	2–25%	12–13.63%	14–18.18%	-	NS
Resynchronization therapy:	10–22.72%	16–29.09%	-	15–13.63%	17–11.97%	3–11.53%	0.002
New implants	3–30%	4–25%	-	6–40%	8–43.75%	3–100%	NS
Implantable cardioverter:	2–4.54%	4–7.27%	1–11.11^	7–6.36%	15–8.73%	5–19.23%	NS
New implants	1–50%	1–25%	0	2–28.57%	5–27.27%	1–20%	NS

Legend: diabetes mellitus—DM; P-patient; *p*—statistical significance; NS—not statistically significant; atrioventricular block—AVB; sick sinus syndrome—SSS; carotid sinus hypersensitivity—CSHS; atrial fibrillation—AF; Statistical methods: Chi-square test, Fisher exact test (Freeman-Halton extension).

## Data Availability

Data are available at https://preventim.ro/public/pages/3.php, accessd on 15 March 2021.
